# Protein Kinase C Alpha is a Central Node for Tumorigenic Transcriptional Networks in Human Prostate Cancer

**DOI:** 10.1158/2767-9764.CRC-22-0170

**Published:** 2022-11-08

**Authors:** Mariana Cooke, Xuyao Zhang, Suli Zhang, Evgeniy Eruslanov, Priti Lal, Reba E. Daniel, Michael D. Feldman, Martin C. Abba, Marcelo G. Kazanietz

**Affiliations:** 1 Department of Systems Pharmacology and Translational Therapeutics, Perelman School of Medicine, University of Pennsylvania, Philadelphia, Pennsylvania.; 2 Department of Medicine, Einstein Medical Center Philadelphia, Philadelphia, Pennsylvania.; 3 Division of Thoracic Surgery, Perelman School of Medicine at the University of Pennsylvania, Philadelphia, Pennsylvania.; 4 Department of Pathology and Laboratory Medicine, Perelman School of Medicine, University of Pennsylvania, Philadelphia, Pennsylvania.; 5 Centro de Investigaciones Inmunológicas Básicas y Aplicadas, Universidad Nacional de La Plata, La Plata, Argentina.

## Abstract

Aberrant expression of protein kinase C (PKC) isozymes is a hallmark of cancer. The different members of the PKC family control cellular events associated with cancer development and progression. Whereas the classical/conventional PKCα isozyme has been linked to tumor suppression in most cancer types, here we demonstrate that this kinase is required for the mitogenic activity of aggressive human prostate cancer cells displaying aberrantly high PKCα expression. IHC analysis showed abnormal upregulation of PKCα in human primary prostate tumors. Interestingly, silencing PKCα expression from aggressive prostate cancer cells impairs cell-cycle progression, proliferation, and invasion, as well as their tumorigenic activity in a mouse xenograft model. Mechanistic analysis revealed that PKCα exerts a profound control of gene expression, particularly over genes and transcriptional networks associated with cell-cycle progression and E2F transcription factors. PKCα RNAi depletion from PC3 prostate cancer cells led to a reduction in the expression of proinflammatory cytokine and epithelial-to-mesenchymal transition (EMT) genes, as well as a prominent downregulation of the immune checkpoint ligand PD-L1. This PKCα-dependent gene expression profile was corroborated *in silico* using human prostate cancer databases. Our studies established PKCα as a multifunctional kinase that plays pleiotropic roles in prostate cancer, particularly by controlling genetic networks associated with tumor growth and progression. The identification of PKCα as a protumorigenic kinase in human prostate cancer provides strong rationale for the development of therapeutic approaches toward targeting PKCα or its effectors.

**Significance:**

PKCα was found to be aberrantly expressed in human prostate cancer. Silencing the expression of this kinase from aggressive prostate cancer cell lines reduces their proliferative, tumorigenic, and invasive properties. In addition, our findings implicate PKCα as a major node for transcriptional regulation of tumorigenic, inflammatory, and EMT networks in prostate cancer, highlighting its potential relevance as a therapeutic target.

## Introduction

Prostate cancer is the second leading cause of cancer-related deaths among men in the United States, with approximately 268,500 new cases and approximately 34,600 projected deaths in the United States for 2022 (1). Although patients with prostate cancer generally respond favorably to available therapies, the disease eventually progresses to metastatic castration-resistant prostate cancer (mCRPC), which is hard to eradicate and is ultimately lethal (2, 3). The initiation and progression of prostate cancer is driven by genetic and epigenetic changes leading to dysregulation of oncogenic signal transduction pathways mostly linked to the acquisition of proliferative and invasive traits. Hallmarks of prostate cancer include the functional inactivation and/or deletion of tumor suppressors (e.g., PTEN, NKX3.1), genomic rearrangements (e.g., TMPRSS2-ERG), altered growth factor signaling (e.g., IGF-1R, FGFR, ErbB receptors), and aberrant signaling (e.g., PI3K/Akt, Src; refs. 4–6).

The protein kinase C (PKC family) has been recognized as a major player in the progression of multiple cancers, including prostate cancer. Members of the three PKC classes (“conventional/classical” cPKCs α, β, and γ; “novel” nPKCs δ, ε, η, and θ; and “atypical” aPKCs ζ and ι) have been widely implicated in fundamental cancer-driving events, such as cell proliferation, survival, motility, and invasion. cPKCs and nPKCs, the PKCs activated by the lipid second messenger diacylglycerol (DAG) and phorbol esters, have a remarkably high functional complexity. Indeed, these kinases could wield either tumor-promoting or tumor-suppressive activities depending on the cell type (7–9). While PKC isozymes are rarely mutated in cancer, upregulation of prooncogenic PKCs and downregulation of tumor-suppressing PKCs are common events in epithelial tumors. This distorted pattern may result in rerouting DAG signals through abnormally overexpressed PKCs, ultimately boosting mitogenic and survival responses through oncogenic signaling pathways such as ERK, NFκB, PI3K, and STAT3 (10). Aberrant PKC isozyme expression has been also linked to epithelial-to-mesenchymal transition (EMT), a dynamic process by which cancer cells acquire invasive properties (11–14). In prostate cancer, PKCε expression is causally associated with disease initiation and progression, as previously established using transgenic mouse models. Conversely, overexpression of other DAG-regulated PKCs in the mouse prostate, namely PKCα and PKCδ, failed to confer a tumorigenic phenotype (15–17). Consistent with these *in vivo* studies, PKCα and PKCδ mediate proapoptotic responses in androgen-dependent prostate cancer cellular models, such as LNCaP cells (18–20). Indeed, there is a vast literature describing PKCα as a tumor-suppressive kinase in several cancers, such as lung, colon, endometrial, and skin cancer (21–26). Intriguingly, PKCα has also been reported to be tumor-promoting kinase, for example in triple-negative breast cancer (26–28). This dichotomy, together with the limited information available in models of prostate cancer, prompted us to investigate the role of DAG-regulated PKCα in this malignancy in further detail.

Here, we show that PKCα is upregulated in primary prostate cancer, and its elevated levels in aggressive prostate cancer cell lines causally associate with proliferative, tumorigenic, and invasive behaviors. We found PKCα to be at the core of gene transcriptional networks associated with the progression of prostate cancer, playing a stringent control over mitogenic, invasive, inflammatory, and tumor evasion gene expression signatures.

## Materials and Methods

### Cell Lines and RNAi Interference

Authenticated human prostate cancer cells were obtained from ATCC and cultured in RPMI medium supplemented with 10% FBS, 2 mmol/L glutamine, 100 U/mL penicillin, and 100 μg/mL streptomycin). Cells are tested for *Mycoplasma* at least twice a year, and normally used at low passage (generally <10 passages).

For the generation of stably depleted PKCα cell lines, we used a short hairpin RNA (shRNA) lentiviral approach. Cells were infected with MISSION shRNA lentiviruses (Sigma-Aldrich) designed for the human *PRKCA* gene (TRCN0000196730, TRCN0000233512), and followed by puromycin selection, as described previously (29).

For transient silencing of PKCα, we used three different siRNA duplexes (#1 and #2 as in ref. 12, #3 from Dharmacon, catalog no. J-003523-18-0005). As non-target control (NTC), we used D-001810-02-05 ON-TARGETplus nontargeting siRNA. siRNA duplexes were transfected using Lipofectamine RNAi Max (Invitrogen), as described previously (30, 31).

### Western Blots

Western blots were done essentially as described previously (31). Briefly, cells were harvested in lysis buffer containing 50 mmol/L Tris-HCl, pH 6.8, 10% glycerol, and 2% β-mercaptoethanol. Cell lysates were subjected to SDS-PAGE and transferred to polyvinylidene difluoride membranes (Millipore Corporation). After blocking with 5% milk or 5% BSA in TBS/0.1% Tween for 1 hour, membranes were incubated overnight with the following primary antibodies: anti-PKCα anti-PKCδ, anti-PKCε (Cell Signaling Technology, catalog nos. 2056, #2058, and #2083, respectively), anti-phospho-Rb (Cell Signaling Technology, catalog no. 2181), anti-caspase-3 (Cell Signaling Technology, catalog no. 9662), anti-caspase-9 (Cell Signaling Technology, catalog no. 9508), anti-PARP1 (Cell Signaling Technology, catalog no. 9532), anti-vimentin (Cell Signaling Technology, catalog no. 5741), anti-Zeb1 (Cell Signaling Technology, catalog no. 3396), anti-AXL (Cell Signaling Technology, catalog no. 8661), anti-E-cadherin (R&D, catalog no. AF748), anti-PD-L1 (Cell Signaling Technology, catalog no. 13684), anti-vinculin (Sigma-Aldrich, catalog no. V9131), or β-actin (Sigma-Aldrich, catalog no. A5441). Membranes were then incubated for 1 hour with either anti-mouse or anti-rabbit secondary antibodies conjugated to horseradish peroxidase (Bio-Rad Laboratories). Bands were visualized and subjected to densitometric analysis using an Odyssey Fc system (LI-COR Biotechnology).

### Cell Proliferation and Viability Assay

Cell number was determined using a Bio-Rad TC20 automated cell counter. Cell viability was assessed using an 3-(4,5-dimethylthiazol-2-yl)-2,5-diphenyl-2H-tetrazolium bromide (MTT) colorimetric assay in 96-well plates. Absorbance at 570 nm was determined in a plate-reader spectrophotometer (EMax Plus Microplate Reader, Molecular Devices Inc.).

### Invasion Assay

A Boyden chamber assay was used, as described previously (17, 29). Briefly, cells were trypsinized, suspended in 0.1% BSA/RPMI, and seeded (2.5 × 10^4^ cells/well) in the upper compartment of a Boyden chamber (NeuroProbe). A 12-μm-pore Matrigel-coated polycarbonate membrane was used to separate the upper and lower compartments. In the lower chamber, RPMI medium containing 10% FBS was used. After an incubation period of 16 hours at 37°C, membranes were recovered and cells on the upper side of the membrane (nonmigrating) were wiped off the surface. Migrating cells on the lower side of the membrane were fixed and stained with the Hema 3 Staining kit (Thermo Fisher Scientific). Migrating cells in each well were counted in five random fields by contrast microscopy using an Eclipse E200 Nikon microscopy (4X magnification) and the ImageJ/Fiji software.

### Tumorigenesis in Nude Mice

Male athymic nude mice (Foxn1^nu^/Foxn1^nu^) were purchased from Harlan Laboratories. Animals were maintained in a temperature-controlled room located at the University of Pennsylvania School of Veterinary Medicine (Philadelphia, PA) and fed *ad libitum*. All animal studies were carried out in strict accordance with the University of Pennsylvania Institutional Animal Care and Use Committee guidelines.

PC3 cells (1 × 10^5^) were injected subcutaneously into the flanks of 6-week-old male athymic nude mice (Foxn1^nu^/Foxn1^nu^). Tumor formation was monitored for 37 days. Tumor volume was determined with caliper measurements and calculated using the formula *L1* × *L2* × *H* × 0.5238, where *L1* is the long diameter, *L2* is the short diameter, and *H* is the height of the tumor.

### RNA Isolation and Real-time Quantitative PCR

Total RNA was extracted from subconfluent plates using the RNeasy kit as directed by the manufacturer (Qiagen), as described previously (31). Briefly, 1 μg of RNA per sample was reverse transcribed using the TaqMan reverse transcription reagent kit and random hexamers as primers (Applied Biosystems). Primers for individual genes were purchased from Applied Biosystems. PCR amplifications were performed using an ABI PRISM 7300 Detection System in a total volume of 20 μL containing Taqman Universal PCR Master Mix (Applied Biosystems), commercial target primers (300 nmol/L), the fluorescent probe (200 nmol/L), and 1 μL of cDNA. PCR product formation was continuously monitored using the Sequence Detection System software version 1.7 (Applied Biosystems). The FAM signal was normalized to endogenous UBC (housekeeping gene).

### RNA Sequencing Data Analysis

RNA was isolated from triplicate subconfluent plates using the RNeasy kit. RNA concentration and integrity were measured on an Agilent 2100 Bioanalyzer (Agilent Technologies). Only RNA samples with RNA integrity values (RIN) over 8.0 were considered for subsequent analysis. One of the triplicate samples in the PKCα2 siRNA group behaved as an outlier based on unsupervised analysis of the transcriptomic data, and therefore this group remained as duplicate samples. RNA samples were processed for directional RNA sequencing (RNA-seq) library construction and sequencing at the Next-Generation Sequencing (NGS) Core facility of the Perelman School of Medicine, University of Pennsylvania (Philadelphia, PA). We performed 100 nt singled-end sequencing using an Illumina HiSeq4000 platform and obtained approximately 20 million reads per sample. The short-sequenced reads were mapped to the human reference genome (hg19) with the splice junction aligner Rsubread R/Bioconductor package (v2.6.3). We employed featureCounts function to calculate the gene expression abundance using the aligned BAM files. To identify differentially expressed genes (log2 fold change > ±1, FDR < 0.001) between the three different siRNA duplexes (α1, α2, or α3) with the NTC siRNA and parental cells, we employed the edgeR R/Bioconductor package based on the normalized log2 based count per million values.

For functional enrichment analyses (FEA), we used ClueGo Cytoscape's plug-in (http://www.cytoscape.org/) and the InnateDB resource (http://www.innatedb.com/) based on the list of dysregulated transcripts. Gene set enrichment analysis (GSEA) of transcription factor binding sites (TFBS) was performed with the R/Bioconductor package clusterProfiler based on the TFBS signature obtained from MSigDB (c3.tft.v7.4.entrez.gmt). RNA-seq expression profiles of 497 primary prostate adenocarcinomas from The Cancer Genome Atlas Prostate Adenocarcinoma (TCGA-PRAD) project were downloaded from the UCSD-Xena resource (https://xena.ucsc.edu/). Prostate carcinomas were divided into low (*n* = 75) or high (*n* = 134) *PRKCA* expression levels according to the StepMiner one-step algorithm for further analysis of PKCα-modulated genes. Data integration and visualization of differentially expressed transcripts were done with R/Bioconductor and the MultiExperiment Viewer software (MeV v4.9).

### Correlation Analyses

To explore associations between PRKCA mRNA levels and EMT phenotype of primary prostate carcinomas, EMTome resource (http://www.emtome.org/) was employed to extract three relevant EMT gene expression signatures identified in invasive prostate carcinomas (32–34). PKCα mRNA expression levels, the EMT gene expression signatures, and their derived EMT scores from TCGA-PRAD dataset were directly retrieved and visualized using the UCSC Xena resource (https://xenabrowser.net/). PKCα mRNA levels and EMT scores for each of the EMT signatures across all primary invasive prostate carcinomas were used for correlation analysis with R software. EMT scores were computed as an average weighted sum of the gene expression levels that constitute each EMT signature.

In a separate association analysis between PKCα expression and androgen receptor (AR), we carried out a meta-analysis of correlation coefficients obtained from eight independent prostate cancer datasets using the DerSimonian-Laird (DSL) random-effect method with correlation coefficients as effect sizes. Briefly, *PRKCA* and *AR* mRNA profiles from primary prostate carcinomas were retrieved from Gene Expression Omnibus (GSE41967 and GSE70768), CancerTool (GSE3325, GSE3933, GSE21032, GSE35988, and MSKCC) and UCSC Xena (GDC-TCGA-PRAD) resources. Pearson correlation coefficients were independently computed from each study in R and subsequently integrated into a meta-analysis using metacor R package.

### Cytokine Measurements

PC3 cells were transfected with siRNA duplexes for PKCα or NTC. After 48 hours, medium was replaced, and conditioned medium (CM) was collected after 12 hours (IL8/CXCL8) or 24 hours (GRO). Cytokines in the CM were determined using the Quantikine ELISA kit for human IL8 (R&D Systems) and the human GRO ELISA kit (Invitrogen), following the protocols provided by the manufacturers.

### Tissue Microarray and IHC

For each case of prostatic adenocarcinoma, one slide most representative of the overall Gleason score and stage was identified. The corresponding paraffin-embedded tissue block was used to construct a tissue microarray (TMA) using 1 mm coring needle (Beecher Tissue Arrayer MTA-1). All 88 cases were represented at least in triplicate resulting in production of four TMA blocks that contained a total of 284 cores. Non-neoplastic liver, kidney, spleen, and tonsil of patients operated for diseases other than cancer was also included in the same blocks as controls. For staining, we used an anti-PKCα antibody (Abcam, ab32376, clone Y124, a validated antibody for IHC in human specimens) at a 1:1,000 dilution. Staining was performed on a Leica Bond-IIITM instrument using the Bond Polymer Refine Detection System (Leica Microsystems DS9800). Heat-induced epitope retrieval was performed for 20 minutes with ER2 solution (Leica Microsystems AR9640). All the experiments were performed at room temperature. For calculation of the *H* score, the intensity of staining was graded on a scale of 0 to 3, with 0 being no staining and 3 being strongest staining. The percentage of tumor cells staining was calculated over multiple high-power fields in the region over the entire tumor. The staining was normalized to compare different tumors by multiplying the intensity of staining with the percentage of tumor cells staining.

### Statistical Analysis

For most experiments, statistical analysis (Student *t* test, ANOVA) was performed using GraphPad Prism software built-in analysis tools. A *P* value < 0.05 was considered statistically significant.

### Data Availability Statement

Raw data for this study were generated at the NGS Core facility of the Perelman School of Medicine, University of Pennsylvania (Philadelphia, PA). Derived data supporting the findings of this study are available in Supplementary Table S1. Other data generated in this study are available within the article and its Supplementary Data.

## Results

### Upregulation of PKCα in Human Prostate Cancer

**FIGURE 1:**
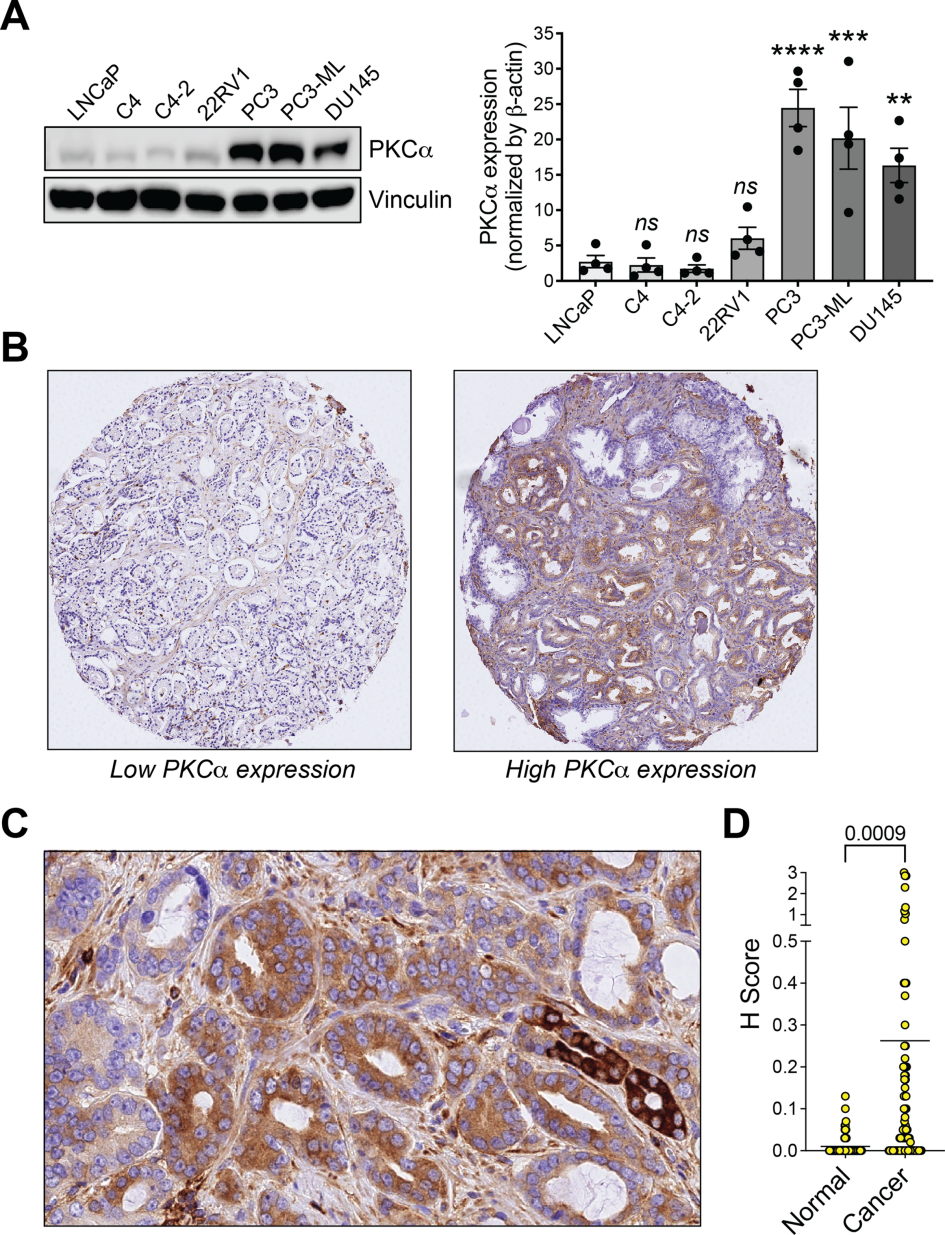
PKCα upregulation in aggressive prostate cancer cells. **A,** Left, PKCα expression in prostate cancer cells, as determined by Western blot analysis. Right, Densitometric analysis. Results are expressed as mean ± SEM (*n* = 4). **B,** Representative IHC staining of PKCα in prostate cancer specimens. Left, Prostate cancer with low PKCα expression. Right, Prostate cancer with high PKCα expression. **C,** Enhanced view of PKCα staining in a prostate cancer specimen. **D,***H* score is plotted for normal and cancer areas stainings with an anti-PKCα antibody. The mean and *P* value are indicated.

The aberrant PKC signaling observed in multiple cancer types foresees important roles for this pathway in disease progression (7, 8), as we previously established for PKCε in prostate cancer (15–17). PKCα, a DAG-responsive PKC with dual effects either as a tumor promoter or suppressor (21–28), has been poorly studied in prostate cancer. Examination of PKCα expression in established prostate cancer cell models showed a prominent upregulation in PC3, PC3-ML, and DU145 prostate cancer cell lines compared with less aggressive LNCaP cells and its sublines (C4, C4-2) or 22RV1 cells (Fig. 1A).

Next, we took advantage of a TMA comprising prostate cancer specimens from 88 patients and evaluated PKCα staining by IHC using an anti-PKCα antibody previously validated in PKCα wild-type versus knockout mice (25), followed by a quantitative analysis reported as *H* score. We observed heterogenous PKCα staining across different specimens, with cases displaying very high staining and others with weak or no staining. Representative examples of TMA micrographs are shown in Fig. 1B. A detailed analysis revealed low or no intensity staining in nontumor areas, whereas PKCα staining was primarily detected in those areas defined as adenocarcinoma. An enhanced view is shown in Fig. 1C. Quantitative analysis of PKCα immunostaining in tumor areas relative to normal areas is depicted in Fig. 1D.

### PKCα Mediates Human Prostate Cancer Cell Growth

**FIGURE 2:**
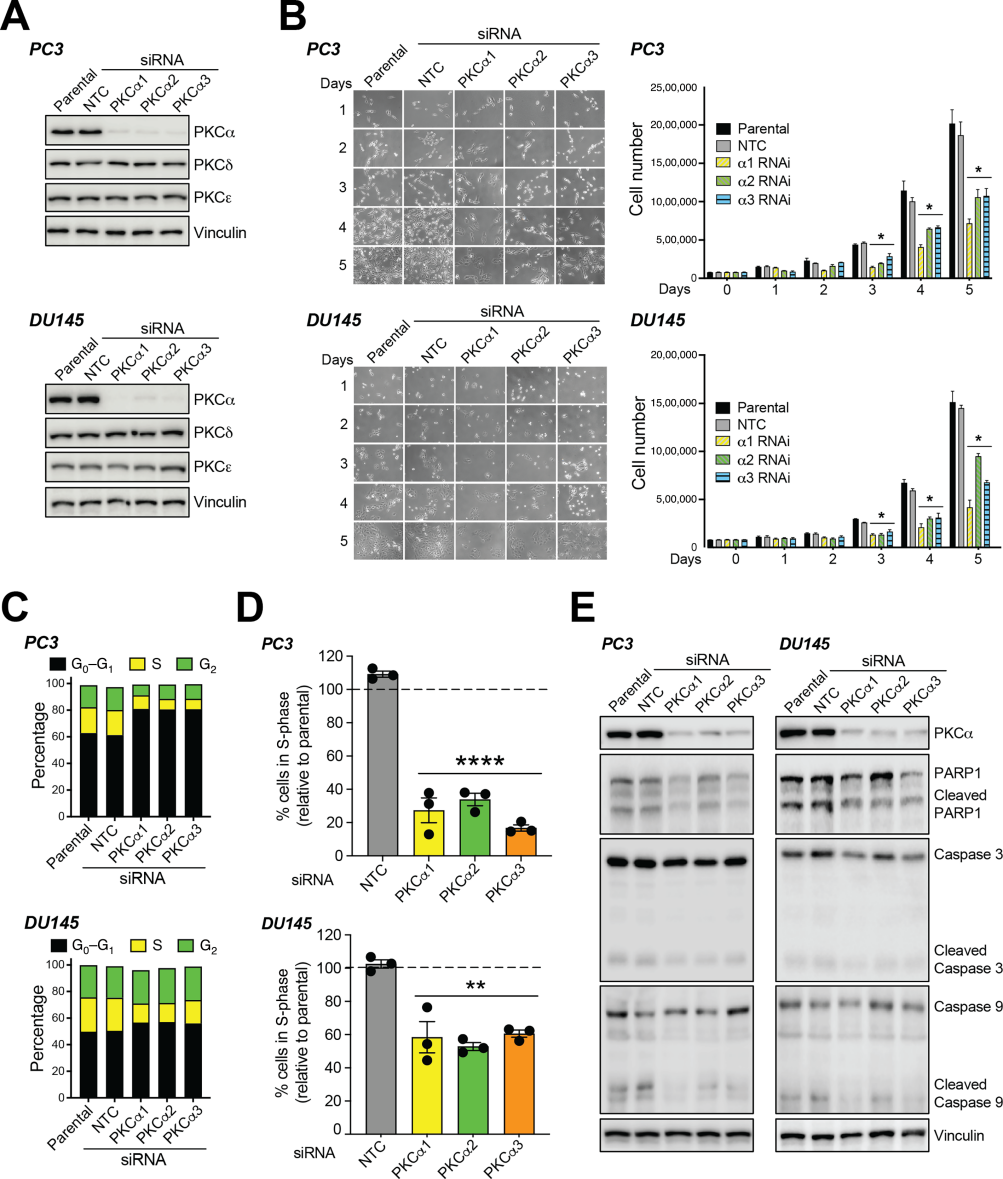
Silencing PKCα from aggressive prostate cancer cells induces cell growth arrest. PC3 or DU145 cells were transfected with three different PKCα (α1, α2, or α3) or *NTC* siRNA duplexes. Twenty-four hours later, cells were serum starved for 24 hours and experiments carried out at the indicated times. P, parental. **A,** Expression of PKC isozymes was determined by Western blot analysis at day 3 after transfection. Representative Western blots are shown. **B,** Effect of PKCα RNAi depletion on cell number. Left, Representative micrographs 1–5 days after transfection of siRNA duplexes. Right, Cell number was expressed as mean ± SD (*n* = 3). An additional experiment gave similar results. **C,** Percentage of cells in S-phase 48 hours after transfection with siRNA duplexes, as determined by flow cytometry in EdU-labeled cells. Results are expressed as mean ± SE (*n* = 3). **D,** Cell-cycle distribution was determined by flow cytometry 48 hours after transfection with siRNA duplexes. Left, Representative flow cytometry charts. Right, Cell-cycle distribution in a representative experiment. **E,** Expression of apoptosis markers (cleaved PARP1, cleaved caspase 3, and cleaved caspase 9). A representative experiment is shown. *, *P* < 0.05; **, *P* < 0.01; ****, *P* < 0.0001 versus NTC.

To begin elucidating the functional consequences of PKCα upregulation in aggressive cellular models of prostate cancers, we first analyzed the effect of PKCα RNAi silencing on proliferation. Three different siRNA duplexes were used to knockdown PKCα in PC3 and DU145 cell lines. These duplexes caused >80% PKCα depletion in all cases without affecting the expression of PKCδ and PKCε, the other DAG-responsive PKCs expressed in these cells (Fig. 2A). PKCα depletion lasted for at least >120 hours posttransfection (Supplementary Fig. S1). Silencing PKCα conferred slower growth rate properties to both PC3 (Fig. 2B, top) and DU145 cells (Fig. 2B, bottom) when compared with parental cells or cells subjected to NTC RNAi. Cell-cycle distribution analysis using FACS revealed an accumulation of PKCα-depleted PC3 and DU145 cells in G_0_–G_1_, with a subsequent reduction in S-phase (Fig. 2C). 5-Ethynyl-2′-deoxyuridine (EdU) incorporation assays showed a significant decrease in the fraction of cells undergoing DNA synthesis in PKCα-depleted PC3 or DU145 cells relative to control cells (Fig. 2D). Consistent with the inhibition in G_1_ to S transition, a prominent Rb dephosphorylation was observed in PKCα knockdown cells (Supplementary Fig. S2A). We were unable to detect any significant changes in the expression of apoptotic markers, namely cleaved PARP1, cleaved caspase-3, and cleaved caspase-9, in PC3 or DU145 cells subjected to PKCα RNAi depletion (Fig. 2E). There was no significant sub-G_0_–G_1_ population in the FACS analysis in PKCα-depleted cells, consistent with the absence of apoptotic cells (Supplementary Fig. S2B). In addition, there was no additive growth inhibitory effect between PKCα RNAi and treatment with either the dual Cdk4/6 inhibitor palbociclib/PD 0332991 (Supplementary Fig. S3A) or the Cdk4 inhibitor NSC 625987 (Supplementary Fig. S3B). Altogether, these results strongly support the requirement of PKCα for mitogenesis in aggressive prostate cancer cells.

**FIGURE 3:**
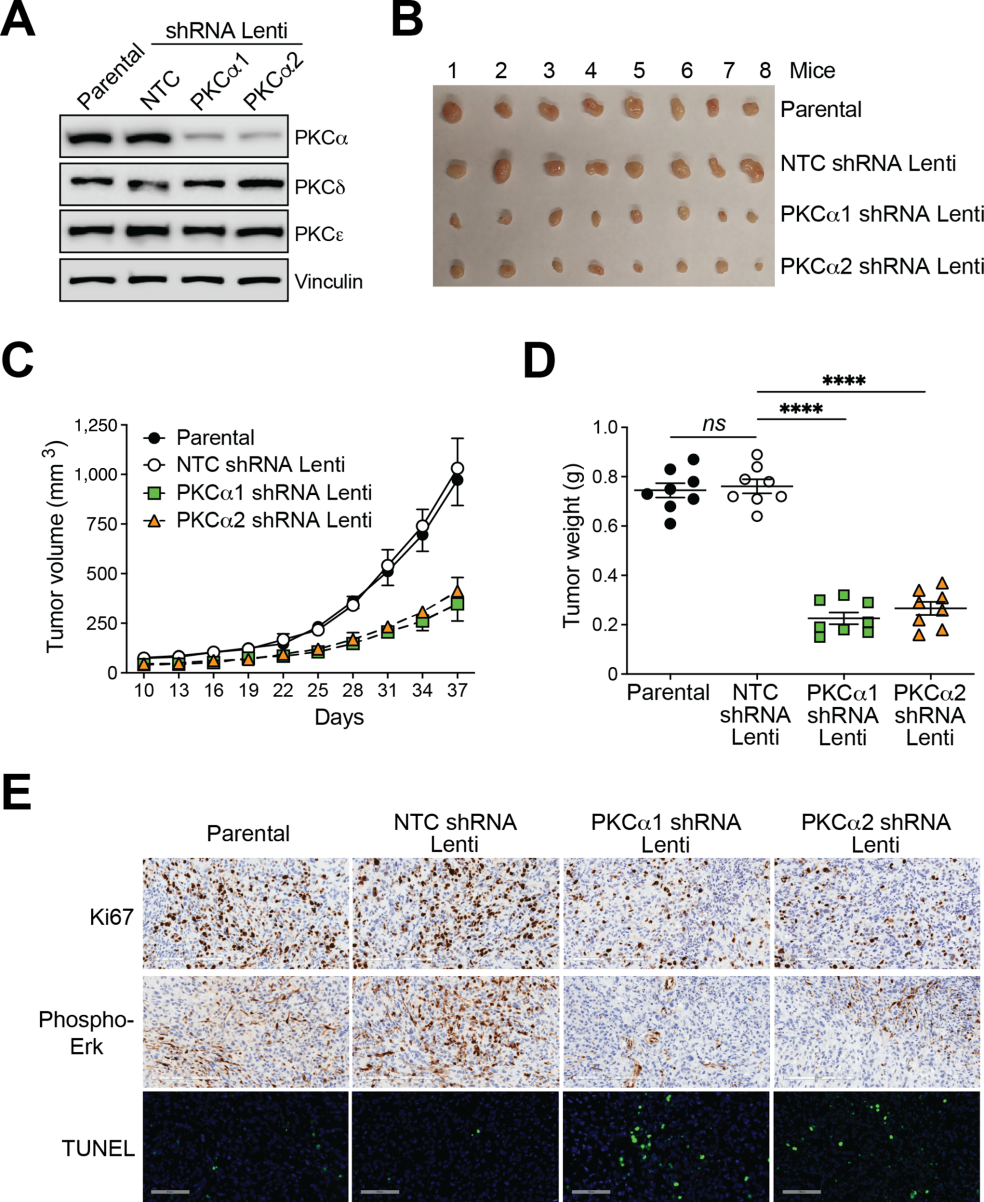
Silencing PKCα reduces PC3 cell tumorigenic activity in nude mice. PC3 cells were subjected to stable PKCα depletion using two different shRNA lentiviruses, followed by puromycin selection. Cells were inoculated subcutaneously into nude mice, and tumor formation was followed for the time indicated in the figure. **A,** Expression of PKC isozymes in the inoculated cell lines, as determined by Western blot analysis. **B,** Pictures of tumors isolated from all mouse cohorts. **C,** Time-course analysis of tumor formation Results are expressed as mean ± SD (*n* = 8). **D,** Tumor weight was determined after sacrificing mice at day 37 postinoculation with the different PC3 cell lines. Results are expressed as mean ± SD (*n* = 8). **E,** Representative IHC images for Ki67, phospho-ERK, and TUNEL. NTC, non-target control shRNA lentivirus. ****, *P* < 0.0001 versus NTC.

Next, to examine whether PKCα is involved in *in vivo* prostate cancer tumor growth, we generated stably depleted PKCα PC3 cell lines using shRNA lentiviruses. Like the siRNA-mediated PKCα transient approach, stable knockdown was highly specific for PKCα without significant changes in the expression of other DAG-regulated PKCs (Fig. 3A). Upon subcutaneous inoculation of parental PC3 cells into nude mice, a tumor growth response was readily observed. Notably, PKCα-depleted PC3 cells displayed a significant reduction in tumor growth compared with parental PC3 cells or cells subjected to NTC RNAi, as determined by measurements of tumor volume (Fig. 3B and C) and weight (Fig. 3D). PKCα-depleted tumors exhibit reduced Ki67 staining, a marker for mitotic index, as well as diminished phospho-Erk staining compared to tumors from parental or NTC PC3 cells. We detected TUNEL-positive areas in PKCα-depleted tumors (Fig. 3E), suggesting that PKCα may confer to some degree an anti-apoptotic response in an *in vivo* setting.

### PKCα Expression Correlates with EMT Markers and is Required for Prostate Cancer Cell Invasion

**FIGURE 4:**
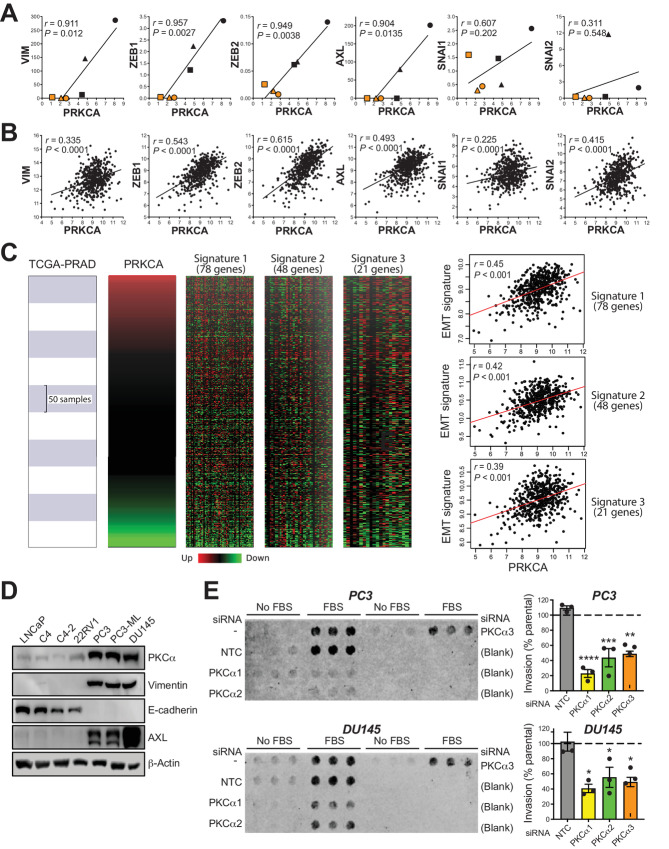
PKCα association with prostate cancer cell line EMT and invasiveness. **A,** Correlations between PKCα (*PRKCA*) and EMT markers from the CCLE database. ●, DU145; ▲, PC3; ■, NCI H660; ●, VCaP; ▲, 22RV1; ■, LNCaP. **B,** Correlations between PKCα (*PRKCA*) and EMT markers from TCGA-PRAD human dataset (498 patients). **D,** Representative Western blot analysis for PKCα and EMT markers in prostate cancer cell lines. **E,** Cell invasion was determined in PC3 and DU145 cells using a Boyden chamber with Matrigel, 48 hours after transfection with siRNA duplexes for PKCα (α1, α2) or NTC. Experiments were done in the absence or presence of 5% FBS in the lower chamber. Left, Representative invasion experiment. Right, Quantification of Boyden chamber migration using 5% FBS. Results are normalized to invasion in parental cells (no siRNA, dotted lines), and expressed as mean ± SEM of three independent experiments. *, *P* < 0.05; **, *P* < 0.01; ***, *P* < 0.001; ****, *P* < 0.0001 versus NTC.

Recent studies linked PKC isozymes to EMT in a variety of tumor types (11–14, 35). We used the Cancer Cell Line Encyclopedia (CCLE) to investigate whether there is an association between the expression of PKCα and markers of the mesenchymal phenotype in prostate cancer cell lines. This analysis revealed a striking positive correlation between the expression of PKCα (*PRKCA* gene) and mesenchymal markers vimentin, ZEB1, ZEB2, and AXL in prostate cancer cell lines (Fig. 4A). A similar positive trend between PKCα and EMT markers was detected in human prostate cancer specimens upon inquire of TCGA-PRAD dataset (Fig. 4B). To further explore the association between *PRKCA* and EMT phenotype, EMTome resource (http://www.emtome.org/) was employed to extract three relevant EMT gene expression signatures identified in prostate carcinomas (32–34). A significant positive correlation between *PRKCA* and prostate cancer EMT signatures was observed in all cases (Fig. 4C). Proof of principle for the association between PKCα expression and EMT was established by Western blot analysis using LNCaP cell lines and 22RV1 as an “epithelial” model and PC3/PC3-ML/DU145 cells as “mesenchymal” models. PC3, PC3-ML, and DU145 cell lines, which aberrantly express PKCα, displayed high expression of mesenchymal markers vimentin and AXL as well as E-cadherin downregulation, whereas the opposite was true for LNCaP/22RV1 cell lines (Fig. 4D).

Because the mesenchymal phenotype of cancer cells is linked to highly invasive traits, we next investigated the effect of knocking down PKCα on prostate cancer cell invasion. Assessment of PC3 and DU145 invasion through Matrigel using a Boyden chamber assay showed a major reduction in transwell migration in PKCα knockdown PC3 and DU145 cells relative to their corresponding parental cells or cells transfected with NTC siRNA (Fig. 4E). Similar results were observed using stably PKCα depleted PC3 and DU145 prostate cancer cells (Supplementary Fig. S4).

Next, we explored a potential relationship between PKCα and AR expression by analyzing eight prostate cancer datasets (see Materials and Methods for details). Although a general pattern could not be defined, a trend for negative correlation was observed in some cases, reaching statistically significance in four datasets. No negative correlation between *PRKCA* and AR expression could be found in TCGA-PRAD. Nonetheless, a meta-analysis of the eight datasets showed a statistically significant negative correlation between *PRKCA* and AR (Supplementary Fig. S5).

### Characterization of the PKCα Transcriptome in Prostate Cancer Cells

**FIGURE 5:**
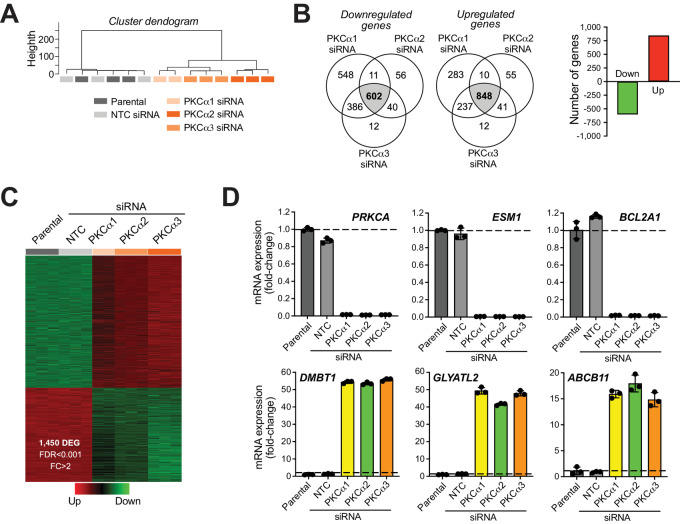
PKCα regulates gene expression in prostate cancer cells. **A,** Dendrogram for deregulated genes in PC3 cells subjected to PKCα silencing using three different siRNA duplexes (α1, α2, or α3), and comparison with NTC siRNA and parental cells. **B,** Number of genes regulated by PKCα, as determined by RNA-seq, which represents the overlapped signature with α1, α2, and α3 siRNA duplexes. **C,** Heatmap for PKCα-regulated genes. **D,** Q-PCR validation the top three upregulated and downregulated genes.

Previous studies from our laboratory established key roles for DAG-regulated PKCs in the control of gene expression (17, 31, 36, 37). To begin elucidating the molecular changes associated with PKCα proliferative and invasive activities in prostate cancer, we carried out a whole transcriptome analysis in PC3 cells. We used five cohorts (parental, NTC siRNA, PKCα siRNA #1, #2, and #3). RNA was extracted 48 hours after transfection of the corresponding siRNA duplexes and subjected to RNA-seq. A comparison of transcriptome profiles was conducted to identify differentially regulated genes using the edgeR test (FDR < 0.001). A 2-fold change relative to parental cells was used as a cutoff. Cluster dendrogram revealed significant overlapping between parental and NTC cells, as well as between the three different PKCα knockdown cell lines (Fig. 5A). Using the described stringent cutoffs, we identified 848 upregulated genes and 602 downregulated genes overlapped by all three PKCα RNAi sequences relative to NTC (*P* < 0.05; Fig. 5B and C). A complete list of PKCα-regulated genes is shown in Supplementary Table S1. As expected, the PKCα gene (*PRKCA*) was a top downregulated gene (>99% depletion), whereas expression of other PKC genes remained unchanged. The AR gene was not among the PKCα-regulated genes (see Supplementary Table S1). Validation of the RNA-seq analysis was done by Q-PCR using specific probes for the three top downregulated genes (*ESM1*, *BCL2A1*, and *PRKCA*) and the three top upregulated genes (*DMBT1*, *GLYATL2,* and *ABCB11*; Fig. 5D).

**FIGURE 6:**
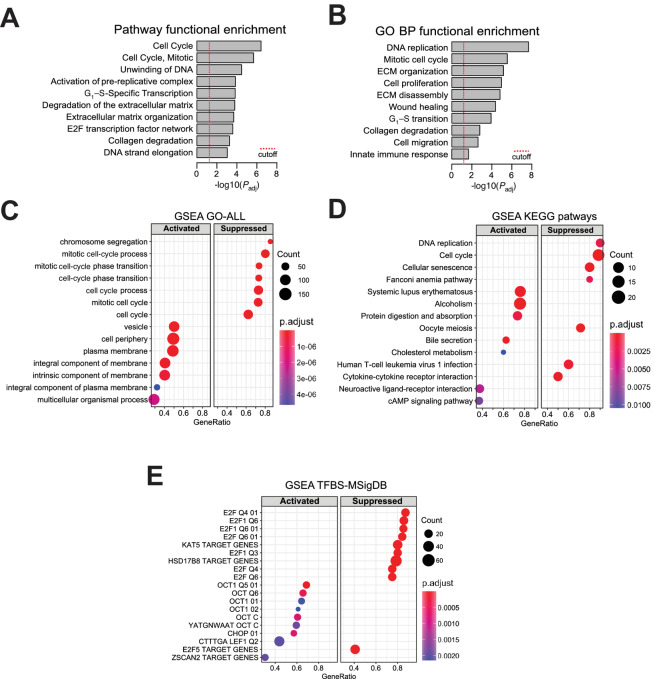
FEAs for PKCα regulates gene expression in prostate cancer cells. **A,** FEA of pathways identified as affected by PKCα silencing in PC3 cells. **B,** FEA of GO biological processes affected by PKCα silencing in PC3 cells. **C,** GSEA of the activated and suppressed bioprocesses in PKCα silencing in PC3 cells. **D,** GSEA of the activated and suppressed KEGG pathways in PKCα silencing in PC3 cells. **E,** GSEA of the activated and suppressed TFBS upon PKCα silencing in PC3 cells based on the TFBS Molecular Signatures Database (MsigDB).

By means of pathway enrichment analysis using REACTOME and Kyoto Encyclopedia of Genes and Genomes (KEGG), we were able to identify prominent enrichment of pathways associated with cell-cycle progression, DNA replication, and regulation of the extracellular matrix (ECM; Fig. 6A). Gene Ontology (GO) analysis provided similar enrichment in gene sets associated with mitogenesis and ECM function, as well as processes related to cell migration and immune responses (Fig. 6B). Complete lists of pathway enrichment analysis and GO enrichment analysis regulated by PKCα can be found in Supplementary Table S2. Further analysis using GSEA for quantitative determination of activated versus suppressed functional enrichments, clearly revealed a major suppression of pathways associated with cell proliferation in PKCα-silenced cells, as depicted in Fig. 6C and D. Top suppressed pathways include “chromosome segregation,” “mitotic cell-cycle process,” “DNA replication,” “mitotic cell-cycle phase transition,” and “DNA replication.”

An analysis of TFBS enrichment in PKCα-regulated genes was performed using the R/Bioconductor package clusterProfiler and the TFBS signatures available at the Molecular Signatures Database. Results depicted in Fig. 6E showed a striking enrichment for E2F TFBSs in the promoters of genes which are downregulated when PKCα is silenced. We also noticed an enrichment for Oct-1 TFBSs in the promoters of genes upregulated upon PKCα silencing. A complete list of transcriptional binding sites regulated by PKCα is presented in Supplementary Table S3. Notably, this E2F signature is suppressed in PKCα knockdown cells, a finding that fits with the expected G_1_ arrest and enhanced E2F activity resulting from Rb dephosphorylation upon PKCα silencing. These results undoubtedly place PKCα as a major hub for the control of transcriptional pathways associated with prostate cancer cell proliferation.

### Differential Expression of EMT Genes in Human Prostate Tumors Based on PKCα Expression

**FIGURE 7:**
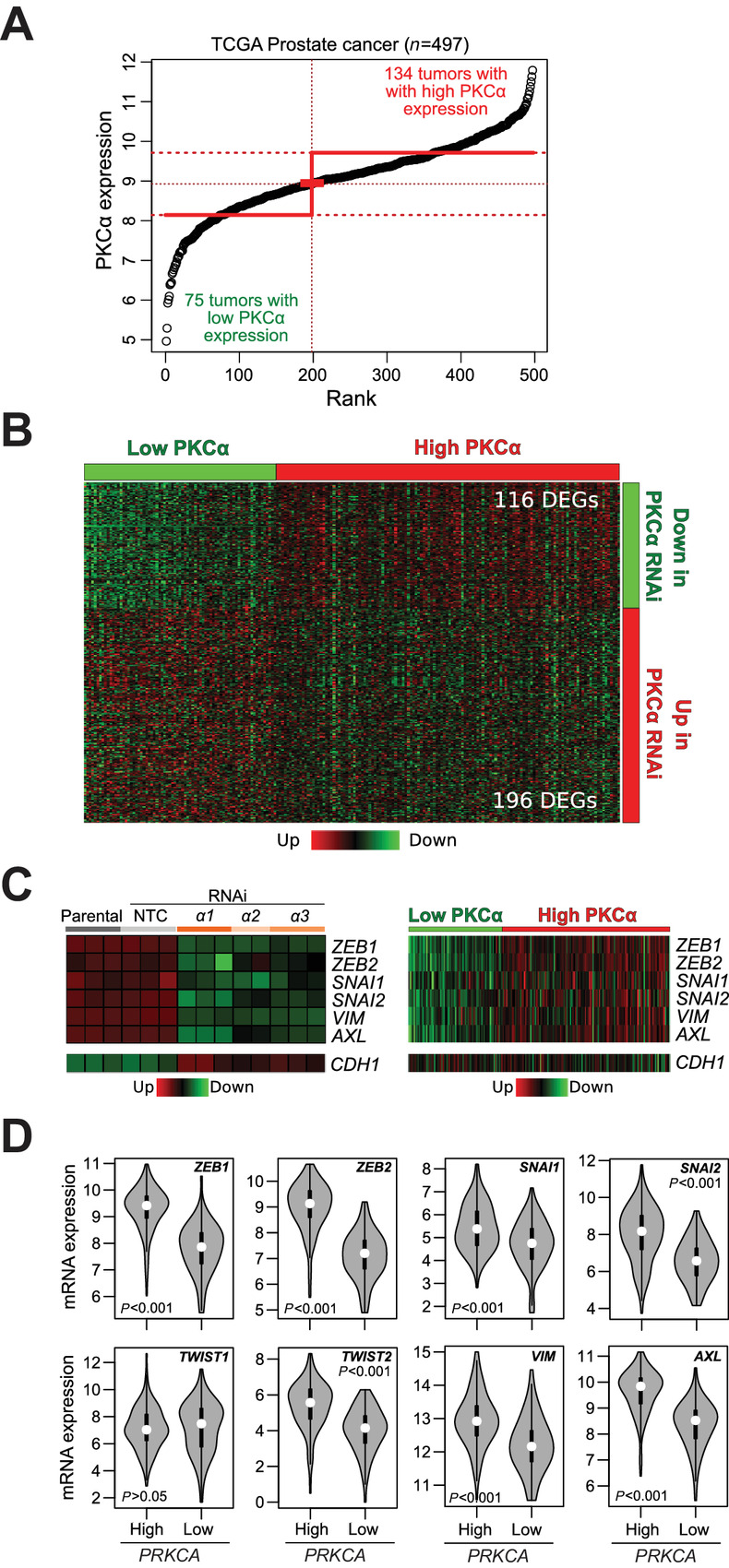
PKCα controls the expression of EMT markers in human prostate cancer. **A,** Classification of primary prostate adenocarcinomas (TCGA-PRAD, *n* = 498) into “*High PKCa*” or “*Low PKCa*” expression levels according to the StepMiner one-step algorithm. **B,** Heatmap showing the differential gene expression from RNA-seq analysis in comparison with the same genes in “*High PKCa*” or “*Low PKCa*” human prostate tumors. **C,** Heatmaps for the expression of EMT-TFs and mesenchymal markers. Left, Expression upon PKCα RNAi silencing in PC3 cells, as determined by RNA-seq. Right, Expression in “*High PKCa*” and “*Low PKCa*” human prostate tumors from TCGA-PRAD. The epithelial marker E-cadherin (CDH1) is also shown. **D,** Violin plots indicating *P* values for the comparison of EMT-TF and mesenchymal marker expression in “*High PKCa*” and “*Low PKCa*” human prostate tumors from TCGA-PRAD.

To further ascertain the biological relevance of the PKCα gene signature identified by RNA-seq in PC3 cells, we next analyzed TCGA-PRAD data collection. Briefly, a group of 498 patients with primary prostate adenocarcinomas were classified into *“Low PKCα”* or *“High PKCα”* mRNA expression levels according to the StepMiner one-step algorithm, establishing 134 tumors and 75 tumors in each category, respectively (Fig. 7A). Differentially expressed genes were scrutinized with the MultiExperiment Viewer software (MeV v4.9), revealing 116 genes in the *“High PKCα”* group which are downregulated in PKCα silenced PC3 cells, and 196 genes in the *“Low PKCα”* group which are upregulated in PKCα-depleted PC3 cells (Fig. 7B). Hence, a significant overlap exists between PKCα-regulated genes in the RNA-seq analysis and a human prostate cancer database.

Next, we carried out an analysis for selected genes associated with epithelial and mesenchymal phenotypes. Interestingly, RNA-seq analysis showed significant downregulation of *ZEB1*, *ZEB2*, *SNAI1,* and *SNAI2*, as also determined in the correlation analysis presented in Fig. 4B. Therefore, PKCα upregulation is associated with the expression of these EMT transcription factors. No changes were observed in *TWIST1*, and *TWIST2* was not detected in the RNA-seq profiling. The RNA-seq in PC3 cells also revealed prominent downregulation of *AXL* and vimentin (*VIM*) upon PKCα silencing (Fig. 7C, left). These results display a remarkable overlap with expression data from TCGA-PRAD, which shows a major upregulation of these EMT markers in *“High PKCα”* human prostate adenocarcinomas relative to the *“Low-PKCα”* patient group (Fig. 7C, right). A significant upregulation of *CDH1* was detected upon PKCα silencing in the RNA-seq profile; however, this was not observed in the human TCGA-PRAD database. Violin plots depicted in Fig. 7D indicate the expression of mesenchymal markers in *“High PKCα”* versus *“Low-PKCα”* groups with the corresponding *P* values.

### PKCα Controls the Expression of Proinflammatory Cytokines and PD-L1 in Human Prostate Cancer

**FIGURE 8:**
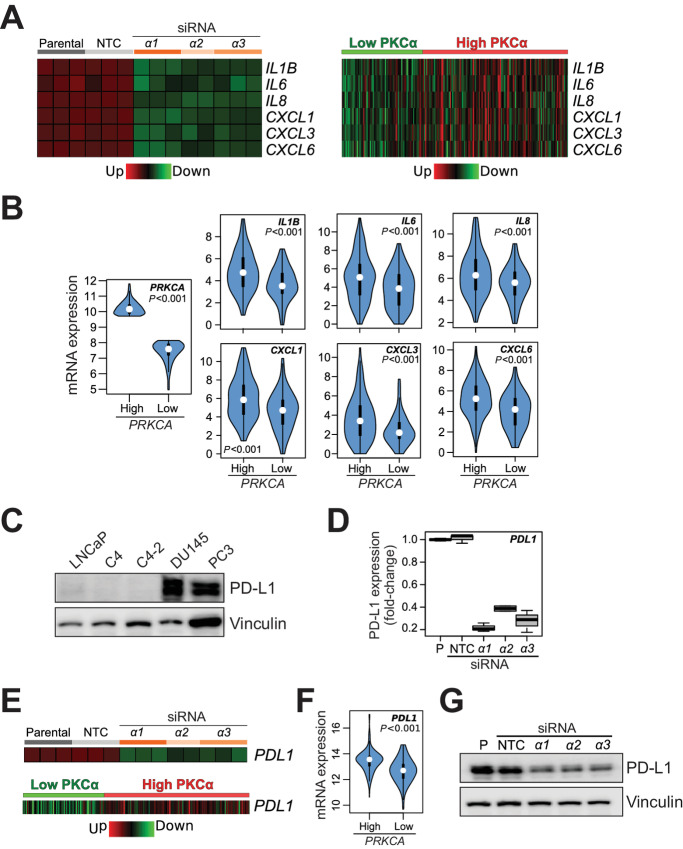
Association of PKCα with proinflammatory and/or tumorigenic cytokines and PD-L1 expression in human prostate cancer. **A,** Heatmaps for the cytokines and/or chemokines regulated by PKCα. Left, Expression upon PKCα RNAi silencing in PC3 cells, as determined by RNA-seq. Right, Expression in “*High PKCa*” and “*Low PKCa*” human prostate tumors from TCGA-PRAD. NTC, non-target control. **B,** Violin plots indicating *P* values for the comparison of cytokine expression in “*High PKCa*” and “*Low PKCa*” human prostate tumors from TCGA-PRAD. **C,** Representative Western blot analysis for PD-L1 expression in prostate cells. **D,** Inhibition of PD-L1 expression in PC3 cells by PKCα RNAi silencing, as determined by RNA-seq. **E,** Heatmaps for PD-L1. Top, Expression upon PKCα RNAi silencing in PC3 cells, as determined by RNA-seq. Bottom, Expression in “*High PKCa*” and “*Low PKCa*” human prostate tumors from TCGA-PRAD. **F,** Violin plots indicating *P* value for PD-L1 expression in “*High PKCa*” and “*Low PKCa*” human prostate tumors from TCGA-PRAD. **G,** PD-L1 protein expression was determined by Western blot analysis in PC3 cells, 48 hours after transfection with siRNA duplexes for PKCα (α1, α2, α3) or NTC.

A focused analysis of cytokine expression from the RNA-seq data revealed prominent downregulation of proinflammatory cytokines in PKCα-depleted PC3 cells, including IL1β, IL6, IL8 (CXCL8), CXCL1 (GRO1), CXCL3 (GRO3), and CXCL6 (Fig. 8A, left). This is remarkable because most of these cytokines have important tumor-promoting roles in prostate cancer and have been associated with high-grade disease (38–40). Interestingly, *“High PKCα”* human prostate tumors displayed elevated expression of these cytokines relative to *“Low PKCα”* tumors, as depicted in heatmaps (Fig. 8A, right) and violin plots (Fig. 8B). Proof of principle for the PKCα-dependent production of cytokines (IL8 and GRO) was determined by ELISA in conditioned medium (Supplementary Fig. S6).

Another important finding from the RNA-seq analysis was the identification of the immune checkpoint PD-L1/CD274 as a PKCα-regulated gene. In agreement with other studies (41, 42), we observed PD-L1 upregulation in PC3 and DU145 cell lines compared to LNCaP cell lines (Fig. 8C). Notably, our RNA-seq analysis showed downregulated PD-L1 mRNA levels in PKCα-silenced PC3 cells, ranging between 60% and 80% (Fig. 8D and E, top). Moreover, analysis in TCGA-PRAD revealed significantly elevated PD-L1 levels in *“High PKCα”* versus *“Low PKCα”* human prostate adenocarcinomas (Fig. 8E, bottom; violin plot in Fig. 8F). We verified the causal association between PD-L1 and PKCα expression at the protein level, with a clear PD-L1 downregulation in PC3 cells upon PKCα RNAi-mediated silencing, as determined by Western blot analysis (Fig. 8G). In conclusion, high PKCα levels associate with upregulation of proinflammatory and/or tumorigenic cytokines as well as with immune checkpoint PD-L1 in human prostate cancer.

## Discussion

Our study established PKCα as a protumorigenic kinase in human prostate cancer. PKCα is prominently upregulated in aggressive cellular models of prostate cancer, and is required for their growth in culture and as xenografts in mice. Using a human prostate cancer TMA, we found PKCα to be highly expressed in a significant fraction of tumors. We hypothesize that abnormally expressed PKCα reroutes DAG inputs by boosting oncogenic PKCα effector pathways, favoring prostate cancer cells to become addicted to this pro-oncogenic axis. The tumorigenic role of PKCα in prostate cancer emphasizes its remarkable functional complexity and epitomizes an example of a DAG/phorbol ester-regulated kinase having dual effects. Indeed, PKCα has been shown to be downregulated and to act as a tumor suppressor kinase in many cancer types. The dichotomous roles of PKCα likely reflect unique links with effector pathways and transcriptional programs depending on expression levels and context, as thoroughly reviewed recently (26), possibly exposing distinctive functional PKCα interactions with oncogenic and tumor-suppressing signals.

Our findings, together with preceding studies, underscore precise roles for PKC isozymes in different stages of prostate cancer progression. We previously generated prostate-specific transgenic mouse models for PKC isozyme overexpression under the control of the probasin (PB) promoter. PB-PKCε mice develop a dysplastic phenotype characteristic of prostatic intraepithelial neoplasia (PIN; ref. 15), underlining a role for PKCε in tumor initiation, as we also postulated in lung cancer models (37). PIN lesions in PB-PKCε mice exhibit early signs of deregulated oncogenic signaling, including hyperactivation of Akt, ERK, STAT3, and NFκB (15–17). However, PB-PKCα mice did not develop any obvious phenotype, indicating its lack of involvement in tumor initiation (15). On the other hand, the PKCα requirement for proliferation strongly supports its involvement in the expanding phase of tumor growth. In this regard, our results show that silencing PKCα expression from PC3 or DU145 cells leads to the accumulation of cells in G_0_–G_1_ and a discernible Rb hypophosphorylation. While the mechanistic aspects of this growth delay remain to be determined, reduced growth upon PKCα inhibition has been linked to p21^cip1^ induction in other cancer types (26, 43–45). The weakened phospho-ERK signal in PKCα-deficient PC3 xenografts fits with the established role of the MEK/ERK cascade in the control of transcriptional events leading to DNA synthesis and mitogenesis (46). Moreover, the enrichment in E2F transcription binding sites in PKCα-regulated genes is a clear indication of the impact that this kinase has on transcriptional events associated with proliferative programs. On the other hand, in scenarios of G_1_ arrest driven by PKCα (i.e., tumor suppression), this kinase drives a transcriptional program of cell-cycle exit, as extensively described by Black and co-workers in models of intestinal epithelial cells (26, 47). In that model, PKCα suppresses the expression of inhibitor of DNA binding (Id) transcription factors through an ERK-dependent mechanism (23, 26). Because Id transcriptions factors have been associated to mitogenesis and oncogenicity, and they are heavily regulated by phosphorylation (48), it would be interesting to determine whether aberrant PKCα signaling contributes to Id activation and/or upregulation, which has been reported in hormone refractory prostate cancer (49, 50). Of note, our RNA-seq analysis reveals a major downregulation of Id1 upon PKCα silencing in PC3 cells (see Supplementary Table S1). Disentangling this enthralling dichotomy represents a significant challenge in PKCα signaling.

Intriguingly, PKCα expression levels do not unequivocally associate with AR responsiveness status. While cell lines displaying aberrant PKCα expression (PC3, PC3-ML, DU145) are AR negative, LNCaP variants that lose androgen responsiveness display low PKCα levels. Notably, PKCα-depleted PC3 cells did not reveal changes in AR levels, an indication that PKCα *per se* does not regulate AR expression. Moreover, analysis of PKCα and AR expression from multiple databases did not show an obvious association, despite a trend for negative correlation in some datasets and a statistically significant negative correlation between *PRKCA* and AR in our meta-analysis. It would be interesting to determine the molecular basis behind the PKCα upregulation in AR negative cells, which presumably involves reprogrammed transcriptional mechanisms that occur in mCRPC.

Our bioinformatics analysis using the CCLE database and the TCGA-PRAD tumor database revealed positive correlations between PKCα and the expression of EMT markers. Notably, *“High PKCα”* tumors express high levels of mesenchymal phenotype markers, including EMT transcription factors. A causal association seems to be in place based on the noted downregulation of these markers upon PKCα silencing, as shown in our RNA-seq analysis. It remains to be determined whether PKCα is causally related to the transition from the epithelial to mesenchymal state in prostate cancer. Our previous studies in lung cancer cells revealed that silencing the expression of individual PKC isozymes, including PKCα, did not prevent the acquisition of the mesenchymal phenotype in response to TGFβ (51). Still, a partial reversal of the mesenchymal phenotype upon PKCα inhibition is seen in breast and lung cancer models (12, 52). A likely possibility is that PKCα upregulation is secondary to the acquisition of a mesenchymal state rather than contributing to the EMT transformation process. Elevated PKCα signaling may aid in the maintenance of the mesenchymal phenotype by upregulating EMT transcription factors (14, 35) and upholding invasive capacity, as revealed in our Boyden chamber analysis using PKCα-silenced cells. Along this line, our previous study in lung cancer models established PKCα as a determining factor for the production of metalloproteases required for ECM degradation (31). PKCα may also control fundamental cellular processes associated with cell migration, a conclusion supported by the enrichment in PKCα-regulated genes associated with cell motility and ECM function. PKCα may regulate basic mechanisms driving cell motility, such as the coordinated processes involved in actin cytoskeleton assembly and disassembly. In fact, PKC signaling regulates Rho GTPases involved in the formation of promotility actin-rich peripheral protrusions (e.g., lamellipodia, ruffles) and stress fibers (29, 30, 49, 53–55). Dissecting PKCα downstream effectors is key to untangle the molecular basis of these regulatory processes.

Finally and unexpectedly, our study identified PKCα as a cancer cell intrinsic factor responsible for PD-L1 upregulation in aggressive prostate cancer cells. It is well known that oncogenes and loss of tumor suppressor genes induce PD-L1 expression in cancer cells, often involving PKC effector pathways such as MEK/ERK (56). Our finding may have significant impact on tumor immune evasion, because elevated PKCα levels in prostate cancer cells may potentially contribute to the abrogation of T-cell antitumor responses. Our RNA-seq analysis also established PKCα as a key signaling node for proinflammatory cytokine expression. Many of the identified cytokines, including IL6, IL8/CXCL8, and IL1β, are known to stimulate cell-autonomous protumorigenic mechanisms and shape the local microenvironment to support growth, survival, and invasion of primary tumors (38–40). An additional inference relates to the ability of PD-L1 to transduce intrinsic signals independent of T-cell PD-1 ligation. Indeed, PD-L1 contributes to the protumoral activities of cancer cells by increasing proliferation and suppressing apoptotic responses. In addition, overexpressed PD-L1 promotes EMT, and augments migratory and invasive properties of cancer cells (57), Thus, our observation raises the question whether a PKCα/PD-L1 link contributes to proliferative and invasive activities of prostate cancer cells in addition to promoting an inflammatory and/or immunosuppressive landscape.

In summary, the identification of PKCα as a “multifunctional” kinase underlines its relevance in the control of myriad of events associated with prostate cancer. Considering the reported antitumorigenic activity of PKC inhibitors, including those targeting cPKCs (10), PKCα may represent an attractive therapeutic target for aggressive prostate cancer. Overall, the extraordinary functional complexity of PKCα signaling merits a dedicated mechanistic analysis in a cancer type–specific manner, determining their eligibility for a rationale pharmacological approach targeting this kinase or its effectors.

## Supplementary Material

Supplementary Figure 1 Time-dependent expression of PKCalpha in prostate cancer cells after RNAi silencing with three different siRNA duplexes.

Supplementary Figure 2 Cell cycle analysis upon PKCalpha RNAi silencing. Representative Rb phosphorylation as well as cell cycle distribution analysis by FACS in PKCalpha silenced PC3 cells are shown.

Supplementary Figure 3 Effect of treatment with Cdk4/6 and Cdk4 inhibitors in combination with PKCα RNAi silencing on prostate cancer cell viability.

Supplementary Figure 4 Effect of PKCalpha depletion using shRNA lentiviruses on PC3 cell invasion, as determined with a Boyden chamber.

Supplementary Figure 5 Relationship between PRKCA and AR expression in prostate cancer. Correlation graphs for individual datasets as well as a meta-analysis are shown.

Supplementary Figure 6 Reduced production of cytokines in PC3 cells subjected to PKCalpha RNAi depletion. IL-8 and GRO were measured in conditioned medium of PC3 cells by ELISA.

Supplementary Table 1 Differentially expressed genes in PKCalpha silenced PC3 cells.

Supplementary Table 2 Pathway and GO enrichment analysis.

Supplementary Table 3 Gene Set Enrichment analysis (GSEA) of transcription factor binding sites (TFBS).
