# Supplementary material for: Protein Kinase C Alpha is a Central Node for Tumorigenic Transcriptional Networks in Human Prostate Cancer
Source: Cancer Res Commun. 2022 Nov 8;2(11):1372–87. doi: 10.1158/2767-9764.CRC-22-0170 (PMC9933888; doi:10.1158/2767-9764.CRC-22-0170)
Supplement: Supplementary Figure 1 — Time-dependent expression of PKCalpha in prostate cancer cells after RNAi silencing with three different siRNA duplexes. [file crc-22-0170-s01.pdf]

Figure S1

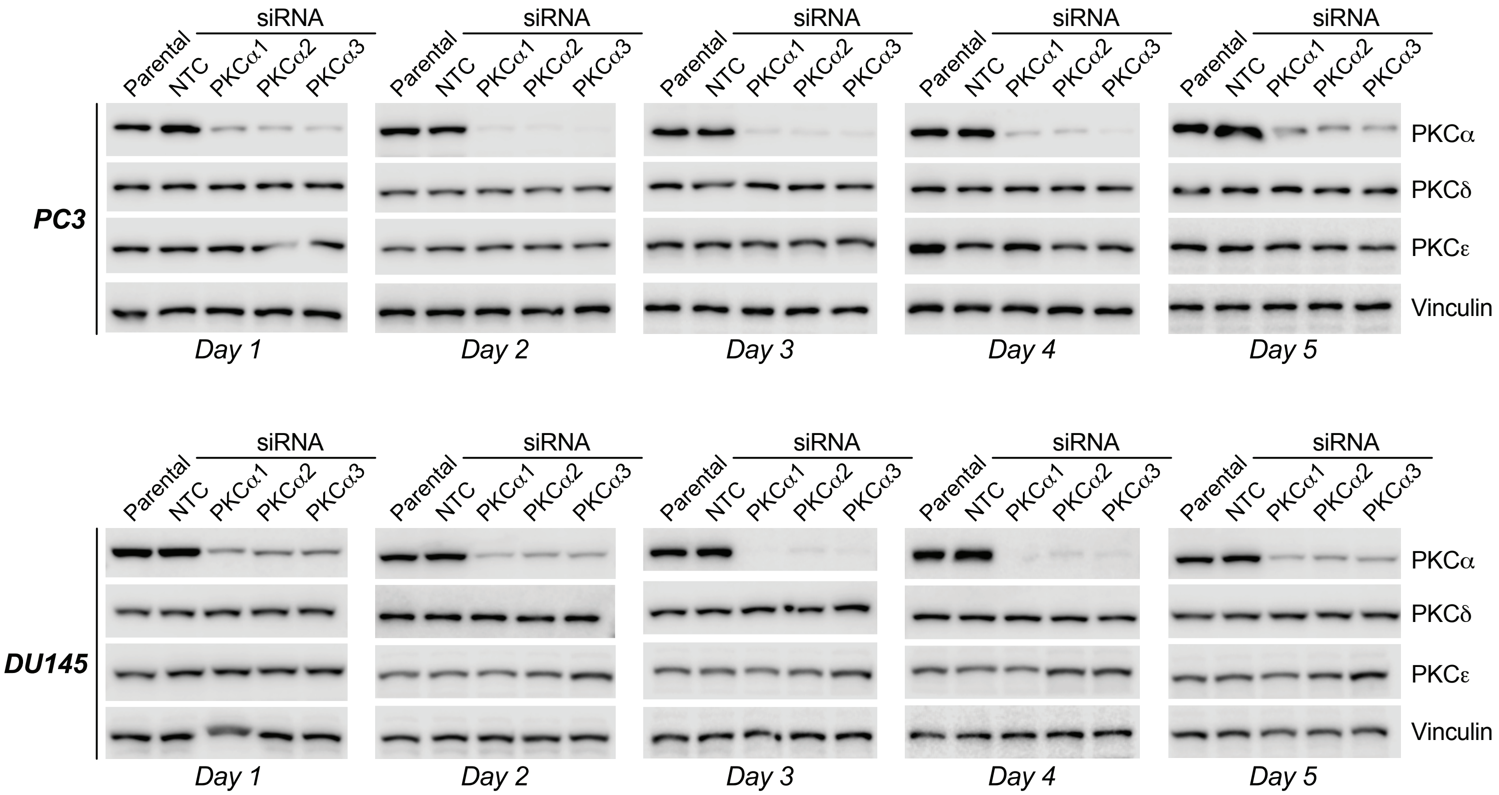

Figure S1

Time-dependent expression of PKCα in prostate cancer cells after RNAi silencing. PC3 or DU145 cells were transfected with three different PKCα siRNA duplexes (α1, α2 or α3) or non-target control (*NTC*). Twenty-four h later cells were serum starved for 24 h, and PKCα expression was determined at different times (1-5 days).
