# Supplementary material for: Protein Kinase C Alpha is a Central Node for Tumorigenic Transcriptional Networks in Human Prostate Cancer
Source: Cancer Res Commun. 2022 Nov 8;2(11):1372–87. doi: 10.1158/2767-9764.CRC-22-0170 (PMC9933888; doi:10.1158/2767-9764.CRC-22-0170)
Supplement: Supplementary Figure 2 — Cell cycle analysis upon PKCalpha RNAi silencing. Representative Rb phosphorylation as well as cell cycle distribution analysis by FACS in PKCalpha silenced PC3 cells are shown. [file crc-22-0170-s02.pdf]

**Figure S2**

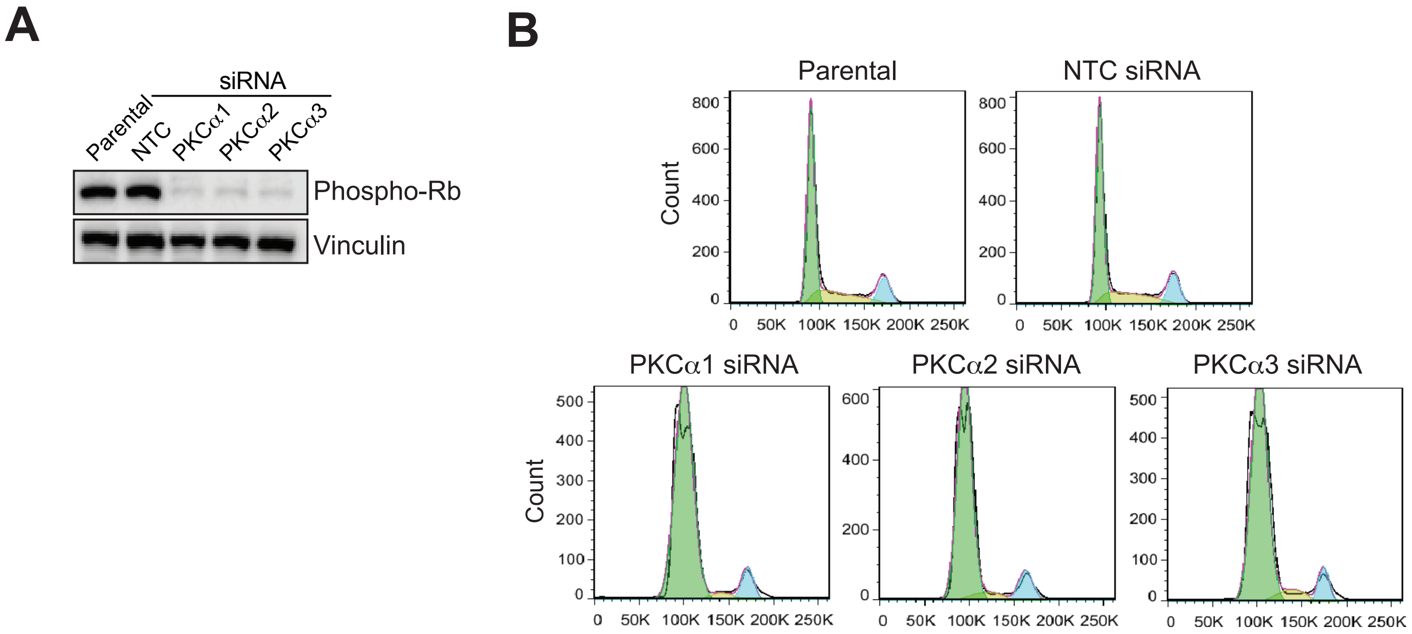


**Figure S2**

Cell cycle analysis. **A,** Rb dephosphorylation in PC3 cells subjected to PKCα RNAi depletion. A representative experiment is shown. **B,** Cell cycle distribution analysis by FACS in PC3 cells, 3 days after transfection with siRNA duplexes for PKCα or non-target control (NTC).
