# Supplementary material for: Protein Kinase C Alpha is a Central Node for Tumorigenic Transcriptional Networks in Human Prostate Cancer
Source: Cancer Res Commun. 2022 Nov 8;2(11):1372–87. doi: 10.1158/2767-9764.CRC-22-0170 (PMC9933888; doi:10.1158/2767-9764.CRC-22-0170)
Supplement: Supplementary Figure 3 — Effect of treatment with Cdk4/6 and Cdk4 inhibitors in combination with PKCα RNAi silencing on prostate cancer cell viability. [file crc-22-0170-s03.pdf]

Figure S3

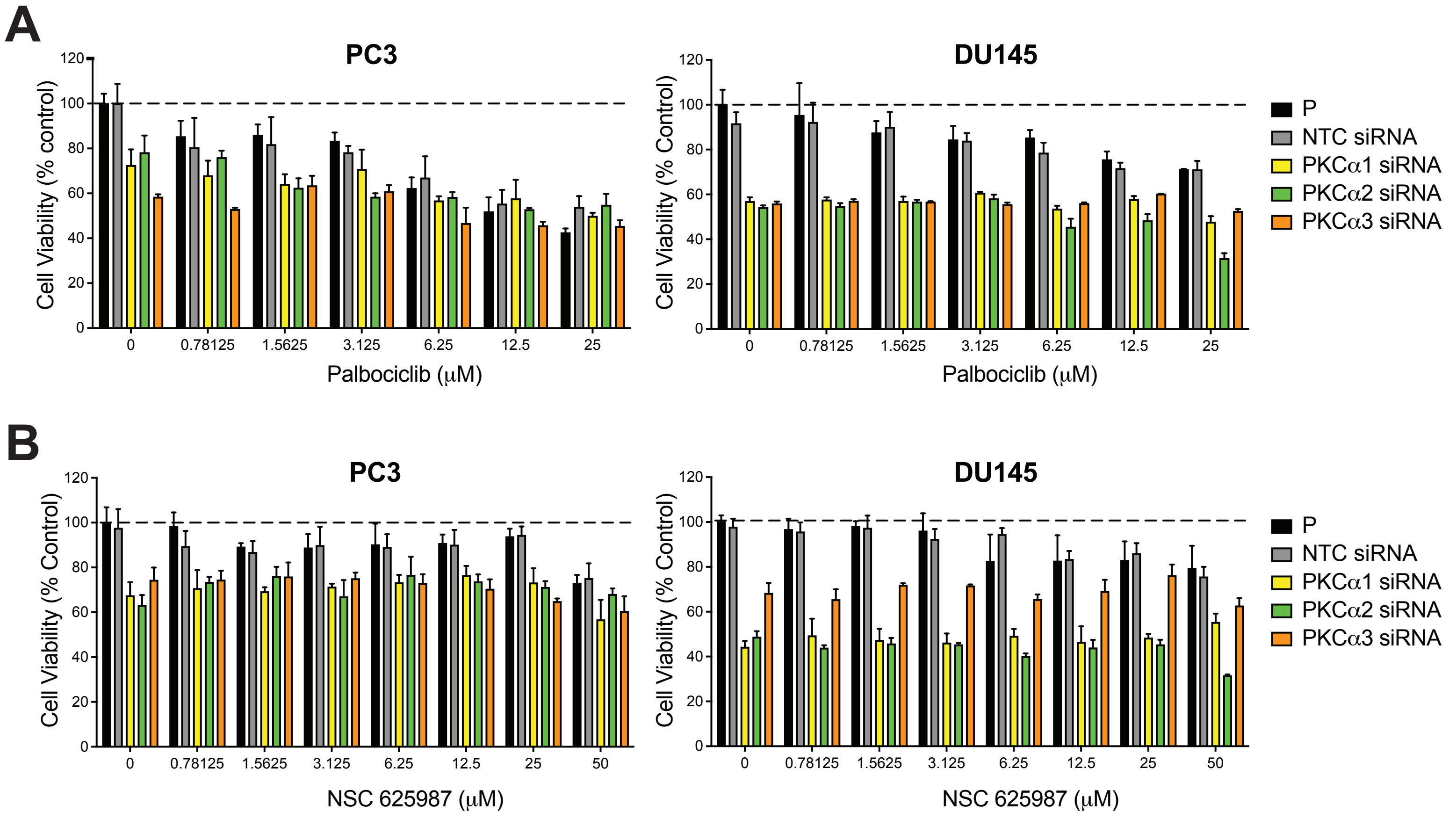

Figure S3

Effect of treatment with Cdk4/6 and Cdk4 inhibitors in combination with PKCα RNAi silencing. **A,** PC3 cells (left panel) or DU145 cells (right panel) were transfected with three different PKCα siRNA duplexes (α1 , α2 or α3) or non-target control (NTC). After 48 h, cells were treated with different concentrations of the Cdk4/6 inhibitor palbociclib for 24 h. Cell viability was determined with the MTT assay. Results (in triplicate) relative to control (parental, non-treated cells) are expressed as mean ± S.D. **B,** Similar experiments as in (A) but using the Cdk4 inhibitor NSC 625987.
