# Supplementary material for: Protein Kinase C Alpha is a Central Node for Tumorigenic Transcriptional Networks in Human Prostate Cancer
Source: Cancer Res Commun. 2022 Nov 8;2(11):1372–87. doi: 10.1158/2767-9764.CRC-22-0170 (PMC9933888; doi:10.1158/2767-9764.CRC-22-0170)
Supplement: Supplementary Figure 4 — Effect of PKCalpha depletion using shRNA lentiviruses on PC3 cell invasion, as determined with a Boyden chamber. [file crc-22-0170-s04.pdf]

Figure S4

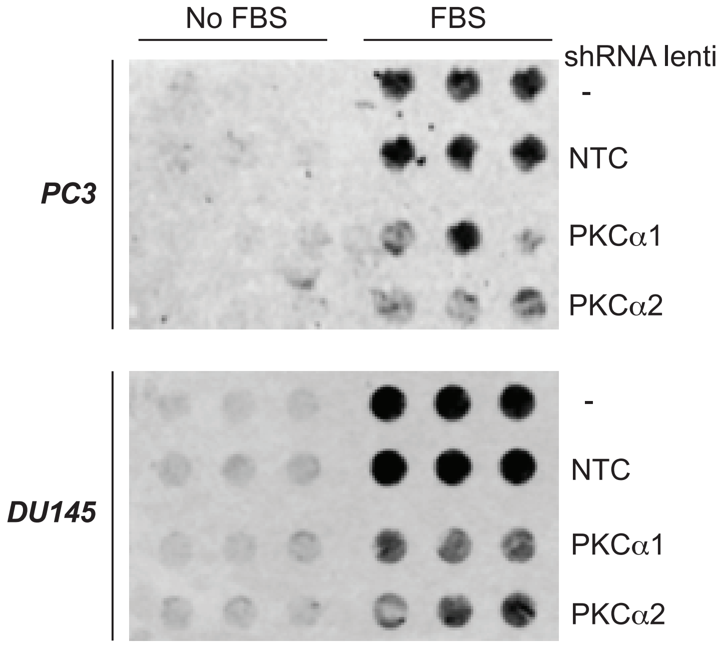

Figure S4

Reduced invasion in PKCα stably depleted prostate cancer cells. Cell invasion was determined using a Boyden chamber with Matrigel in PC3 and DU145 cells subjected to stable PKCα depletion with shRNA lentiviruses. Experiments were done in the absence or presence of 5% FBS in the lower chamber. *NTC*, non-target control shRNA lentivirus.
