# Supplementary material for: Protein Kinase C Alpha is a Central Node for Tumorigenic Transcriptional Networks in Human Prostate Cancer
Source: Cancer Res Commun. 2022 Nov 8;2(11):1372–87. doi: 10.1158/2767-9764.CRC-22-0170 (PMC9933888; doi:10.1158/2767-9764.CRC-22-0170)
Supplement: Supplementary Figure 5 — Relationship between PRKCA and AR expression in prostate cancer. Correlation graphs for individual datasets as well as a meta-analysis are shown. [file crc-22-0170-s05.pdf]

Figure S5

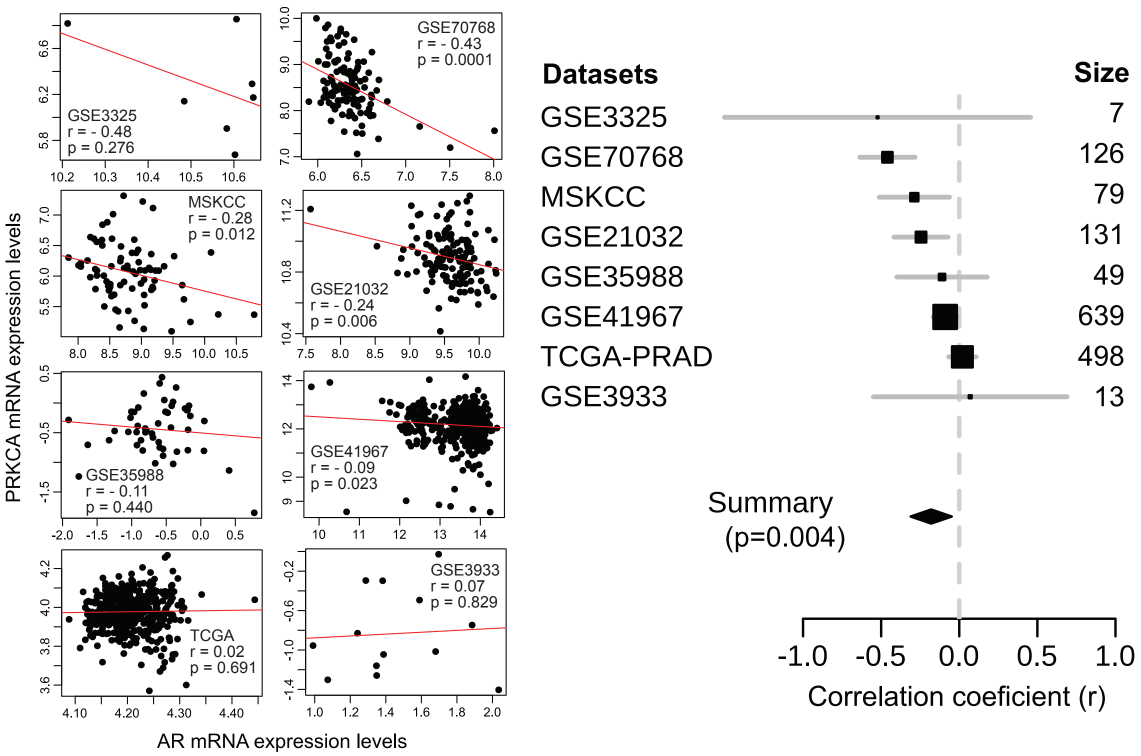

Figure S5

Relationship between *PRKCA* and *AR* expression in prostate cancer. Correlation graphs for individual datasets, including the corresponding *r* and *p* values, are displayed on the left. A meta-analysis is shown on the right.
