# Supplementary material for: Protein Kinase C Alpha is a Central Node for Tumorigenic Transcriptional Networks in Human Prostate Cancer
Source: Cancer Res Commun. 2022 Nov 8;2(11):1372–87. doi: 10.1158/2767-9764.CRC-22-0170 (PMC9933888; doi:10.1158/2767-9764.CRC-22-0170)
Supplement: Supplementary Figure 6 — Reduced production of cytokines in PC3 cells subjected to PKCalpha RNAi depletion. IL-8 and GRO were measured in conditioned medium of PC3 cells by ELISA. [file crc-22-0170-s06.pdf]

Figure S6

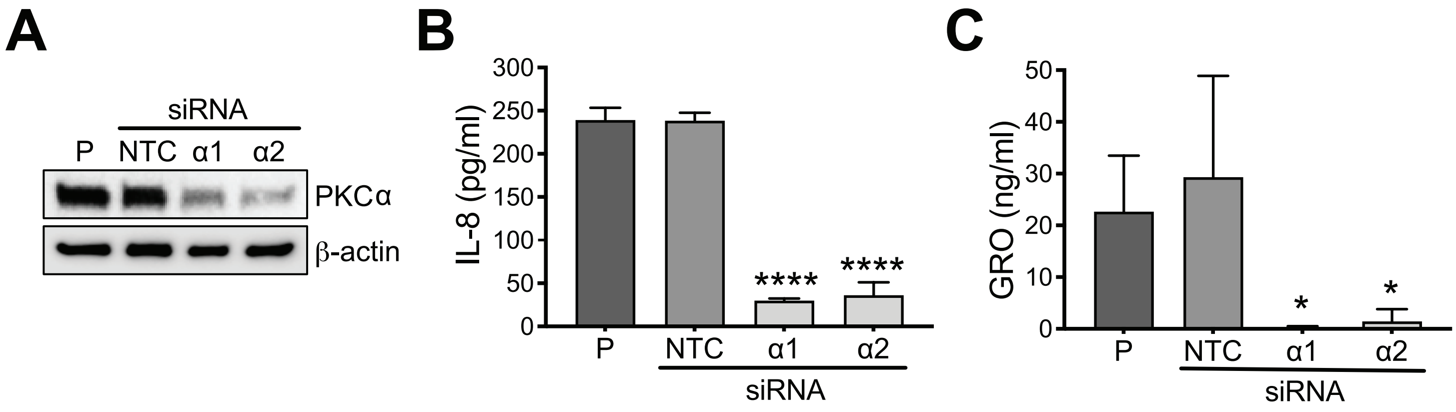

Figure S6

Reduced production of cytokines in PC3 cells subjected to PKCα RNAi depletion. PC3 cells were transfected with PKCα (α1, α2) or non-target control (*NTC*) siRNA duplexes. After 48 h, medium was replaced, and conditioned medium (*CM*) was collected after 12 h (IL-8/CXCL8) or 24 h (GRO). Cytokines in the CM were determined by ELISA. *, $p<0.05$ *vs.* NTC; ****, $p<0.0001$ *vs.* NTC. **A,** Expression of PKCα by Western blot. **B,** Cytokine determination by ELISA. A representative experiment is shown, with data expressed as mean ± S.D. (n=4). *P*, parental.
